# Intragenic suppressors of the *C. elegans* *clk-2(qm37)* mutation

**DOI:** 10.17912/micropub.biology.001828

**Published:** 2025-10-02

**Authors:** Marin Pascal, Laurent Cappadocia, Siegfried Hekimi, Claire Y. Bénard

**Affiliations:** 1 Université du Québec à Montréal, Montreal, Quebec, Canada; 2 Chemistry, Université du Québec à Montréal, Montreal, Quebec, Canada; 3 Biology, McGill University, Montreal, Quebec, Canada; 4 Biological Sciences, Université du Québec à Montréal, Montreal, Quebec, Canada

## Abstract

The *C. elegans* gene *clk-2* , which encodes the orthologue of yeast Tel2p and human TELO2, is implicated in development, lifespan, and telomere length regulation, among other functions. The thermosensitive allele *clk-2 ( qm37 )* (C772Y) is lethal and sterile at 25 °C. We identified eight intragenic mutations that rescue *qm37* defects in suppressor screens. Six yield an A828T substitution, one A828V, and one S859N. All restore viability and fertility at 25 °C, with A828T conferring the most efficient suppression at higher temperature. AlphaFold modeling of the proteins encoded by *clk-2 ( qm37 )* and suppressor mutations points to suppressors stabilizing the CLK-2 (C772Y) protein, and suppression correlates with increased protein accumulation.

**Figure 1. Molecular characterization of clk-2(qm37) intragenic suppressors:**
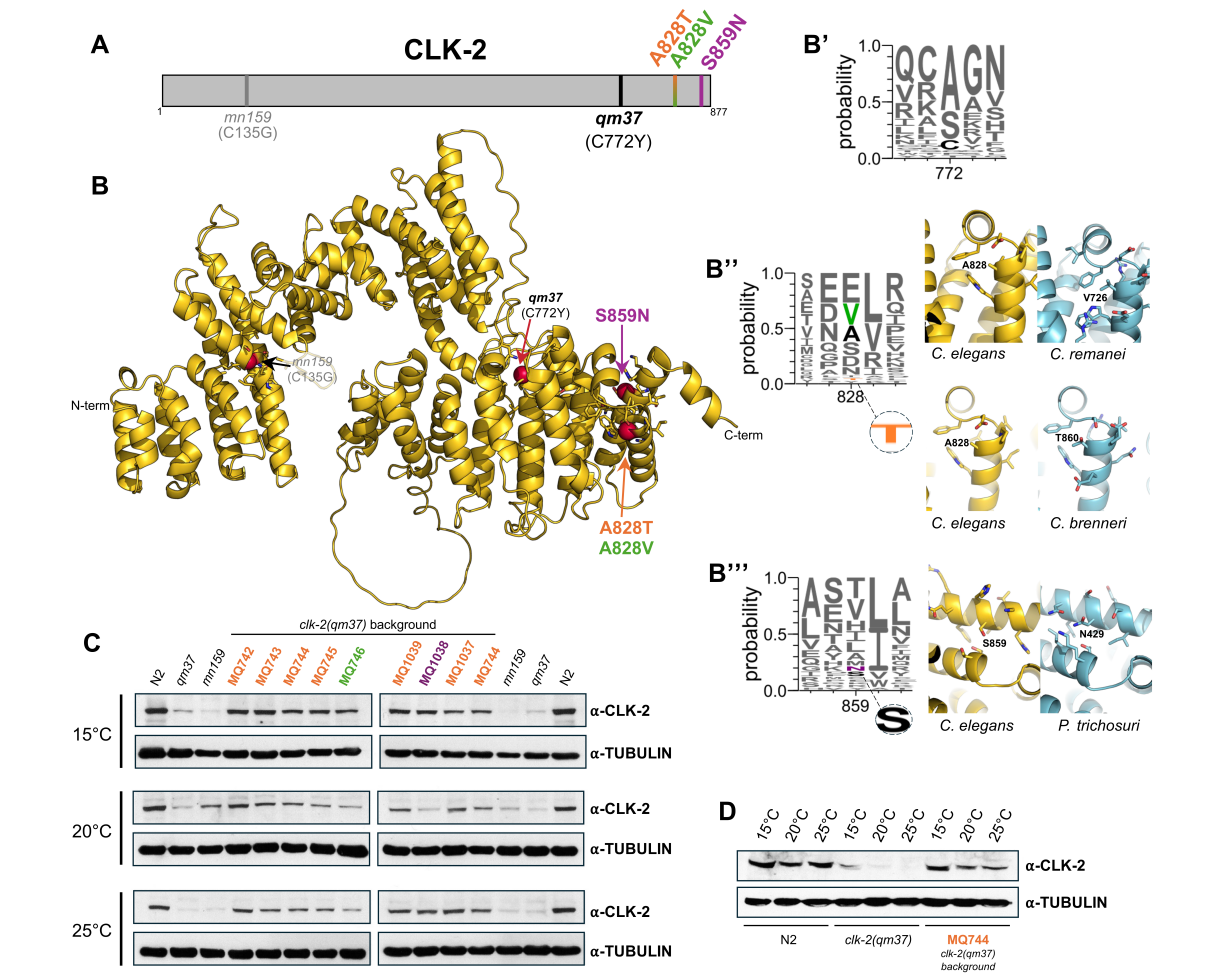
(A) The *clk-2 ( qm37 )* mutation leads to a cysteine to tyrosine substitution at residue 772 of the CLK-2 protein. Among the eight isolated intragenic suppressors, six result in an alanine to threonine substitution at residue 828 (orange; strains MQ742 , MQ743 , MQ744 , MQ745 , MQ1039 , MQ1037 ); one introduces an alanine to valine substitution at residue 828 (green, MQ746 ); and one produces a serine to asparagine substitution at residue 859 (purple, MQ1038 ). (B) Predicted overall structure of CLK-2 protein using AlphaFold (accession Q95YE9) and depicted in cartoon representation. The *qm37* and *mn159* alleles, as well as the intragenic suppressors, are shown with red spheres. Conservation of residues structural position of C772 ( **B'** ), A828 ( **B''** ), and S859 ( **B'''** ) is illustrated by sequence logos generated with WebLogo 3.7.12 from alignments of 54 nematodes CLK-2 homologs. The local structural environments of these residues are shown for *C. elegans* CLK-2 protein and homologs from *C. brenneri* (accession B6VBE2) *,* *C. remanei* (accession A0A260Z4C7), and *P. trichosuri* (accession A0A0N4ZUU2), which naturally contain the same residues as the identified suppressors. Mutated residue positions are shown in stick representation together with neighboring residues. Structures used in the structural alignment were obtained from the AlphaFold database. (C) Western blot analysis of CLK-2 protein in *C. elegans* extracts. CLK-2 is detected at ~100 kDa, α-tubulin at ~50 kDa. CLK-2 protein levels are drastically reduced in *clk-2 ( qm37 )* and *clk-2 ( mn159 )* compared to the wild type at 15, 20, and 25°C. Intragenic suppressors partially restore CLK-2 levels at all temperatures. (D) The A828T suppressor only partially restores protein levels at all temperatures, and similar to the *clk-2 ( qm37 )* mutants, there is a greater accumulation of CLK-2 (C772Y-A828T) at lower than at higher temperatures.

## Description

The *C. elegans* maternal-effect gene *clk-2* encodes an ortholog of the *S. cerevisiae* telomere length regulator Tel2p and human TELO2, implicated in the You-Hoover-Fong syndrome (Albokhari et al., 2023). In worms, *clk-2* affects embryonic and post-embryonic development, reproduction, rhythmic behaviors, and lifespan (Hekimi et al., 1995; Lakowski and Hekimi, 1996; Bénard et al., 2001; Moser et al., 2009), and also participates in DNA damage checkpoint signalling (Ahmed et al., 2001) and nonsense-mediated mRNA decay (Guo et al., 2021). The *clk-2 ( qm37 )* allele is a maternal-effect thermosensitive mutation (C772Y substitution) (Bénard et al., 2001) that is viable at 15 and 20°C, but causes embryonic lethality and sterility at 25°C.

To identify suppressors of *clk-2 ( qm37 )* lethality at 25°C, we screened ~200 000 haploid genomes in the F2 generation and ~110 000 haploid genomes in the F3 generation, isolating eight suppressor mutations (namely, *qv36* , *qv37* , *qv38* , *qv39* , *qv40* , *qv41* , *qv42* , and *qv43* ). All eight were kept at 25°C, outcrossed with *dpy-17 ( e164 ) clk-2 ( qm37 )* III, and found to be dominant and linked to LGIII. Two mapped to a 0.5 cM interval of LGIII between *sma-3* and *unc-36* by three-factor mapping, suggesting intragenic suppression. Sequencing of the *clk-2* locus confirmed all eight as intragenic suppressors, as well as in the remaining 6 suppressors. All 8 suppressors were found to be intragenic transitions (G → A, or C → T). Six suppressors caused an A828T substitution, one an A828V substitution, and one a S859N substitution ( **Fig. 1A** ).

All three classes of suppressors completely suppressed the embryonic lethality and sterility at 25 °C, as well as the slow development at 20°C, and prolonged defecation cycles at 15 and 20 °C seen in *clk-2 ( qm37 )* mutants. At 25 and 26.5 °C, A828T conferred stronger suppression than A828V and S859N. For example, defecation cycle lengths averaged at 47.1 s for the wild type, 49.7 s for A828T, 64.9 s for A828V and 60.0 s for S859N. When L1 larvae are shifted from 20 °C to 26.5 °C, *clk-2 ( qm37 )* mutants develop into scrawny sterile adults, whereas suppressor strains produced large adults with wild-type fertility in the case of A828T, while A828V and S859N remained sterile. At 27 °C, suppression was incomplete: although less affected than *qm37* , suppressor strains developed into large sterile adults, while wild type remained fertile and *qm37* mutants arrested as L3/L4 larvae.

To investigate the molecular basis of suppression, we modelled the effects of *clk-2 ( qm37 )* and suppressor mutations using sequence alignment and AlphaFold predictions ( **Fig. 1B** ). Residue C772 is poorly conserved among nematodes ( **Fig. 1B '** ), often replaced by similar or smaller amino acids (e.g., serine, alanine), suggesting selective pressure against bulky amino acids in this region. Residues at positions 828 ( **Fig. 1B ''** ) and 859 ( **Fig. 1B '''** ) vary across related species and are spatially distant from C772, implying that suppression likely acts by stabilizing the protein rather than directly compensating for C772Y.

Strikingly, orthologous proteins in related species contain the same residues as those present in the *clk-2 ( qm37 )* suppressors. For example, in *C. brenneri* ( **Fig. 1B ''** ), threonine occupies the equivalent structural position to A828T of *C. elegans* CLK-2 (C772Y-A828T). Its methyl group could interact with the aromatic groups of phenylalanine 824 and tryptophan 832, while its hydroxyl group may form hydrogen bonds with aspartic acid 825, potentially reinforcing local structure. Similarly, in *C. remanei* , the ortholog carries a valine at this same structural position, mimicking the A828V suppressor; the two additional methyl groups could reinforce the local structure by interacting with the aromatic groups of phenylalanine 824 and tryptophan 832.

Likewise, in the nematode *P. trichosuri* , the CLK-2 ortholog carries asparagine at the structural equivalent of residue 859, paralleling the S859N suppressor ( **Fig. 1B '''** ). In *C. elegans* , an asparagine at position 859 could form hydrogen bonds with histidine 858/lysine 862 and with threonine 812, further stabilizing the local structure of the CLK-2 (C772Y-S859N) protein. Thus, structural comparisons among homologs reveal that suppressor residues exist in natural contexts, where they may contribute to alternative stabilizing interactions. These observations support a model in which intragenic suppressors enhance the stability of the otherwise unstable CLK-2 (C772Y) protein by reinforcing local structure stability.

Consistent with this, immunoblot analyses revealed that CLK-2 protein levels are higher in suppressors than in *clk-2 ( qm37 )* mutants, but remain lower than in the wild-type at all temperatures ( **Fig. 1C and D** ). In particular, CLK-2 (QM37) protein is undetectable at 25°C, whereas CLK-2 (C772Y-A828T), CLK-2 (C772Y-A828V), and CLK-2 (C772Y-S859N) proteins accumulate robustly. Protein levels in suppressors decreased with temperature ( **Fig. 1C** ). Although protein levels in A828T were not significantly higher than A828V and S859N ( **Fig. 1C** ), A828T suppressed phenotypes more efficiently.

Together, these findings indicate that suppression of *clk-2 ( qm37 )* arises from second-site substitutions that stabilize otherwise unstable CLK-2 (C772Y) protein, allowing accumulation of partially functional protein. Among them, A828T provides the most effective suppression, highlighting how subtle amino acid substitutions can differentially restore protein stability and function.

## Methods

**Isolation of suppressors of the embryonic lethality of the *clk-2 ( qm37 )* mutants at 25°C.** *clk-2 ( qm37 )* mutants maintained at 20°C and mutagenized with 25 mM ethyl methane sulfonate (EMS) as described (Brenner, 1974). Mutagenized worms were washed with M9 and plated onto plates. Two hours later, five young adults (P0s) were transferred to 9 cm Petri dishes, allowed to self-fertilize at 20 °C, and removed after they had laid ~150 embryos in total. F1 progeny were grown at 20 °C until most reached the L2-L3 stages, then plates were shifted to 25 °C to allow completion of larval development. F2 broods were examined 3-4 days later. Most plates contained scrawny, sterile F1 animals that produced few dead F2 embryos. Rare plates carried fertile worms that produced live F2 progeny at 25 °C; these were retained as candidate *clk-2 ( qm37 )* suppressors for further analysis.

In a separate screen, F1 progeny were allowed to develop and self-fertilize at 20 °C until most F2 animals had reached the L2-L3 stages. Plates were then shifted to 25 °C for completion of F2 larval development. F3 broods were examined 3-4 days later. Most plates contained scrawny, sterile F2 worms producing few dead F3 embryos. Rare plates carried fertile worms that yielded live F3 progeny, which were retained for further analysis.

**Genetic analysis of the *clk-2 ( qm37 )* suppressor mutations.** Males for each of the eight suppressors were generated by heat shock and mated into *dpy-17 ( e164 ) clk-2 ( qm37 )* hermaphrodites at 20 °C. For each outcross, 12 L4 non-Dpy F1 cross progeny were singled and shifted to 25 °C. The F1s were invariably fertile and produced live broods, indicating that the suppressors were dominant. Among F2 progeny, 16 Dpy F2 hermaphrodites were singled at 25 °C and found to be sterile, indicating that the suppressor mutations were linked to LGIII. Also, 16 L4 non-Dpy F2 hermaphrodites were singled at 25°C; ~1/3 of them produced entirely wild-type broods, consistent with suppressor homozygosity. Once the molecular nature of each mutation was identified, all eight suppressors were outcrossed 3-5 times with *dpy-17 ( e164 ) clk-2 ( qm37 )* .

**Prediction and analysis of the three-dimensional structure of CLK-2** . The three-dimensional structure of CLK-2 (Uniprot accession Q95YE9) was obtained from the AlphaFold Database (model corresponding to accession Q95YE9 was created on May 31 2022 with the AlphaFold Monomer v2.0 pipeline) (Jumper et al., 2021; Varadi et al., 2022). The images of the structure were made using PyMOL 3.1.3. Sequence logos were generated with WebLogo 3.7.12, using alignments of 54 protein sequences from nematode species and generated by FoldSeek.

**Immunoblotting**

Protein extracts were prepared from mixed-stage populations as described (Bénard et al, 2001), and 50 µg were loaded per lane. Western blotting was performed using primary antibody MG19 rabbit anti- CLK-2 antibodies (Bénard et al, 2001), and HRP-conjugated goat anti-rabbit IgG (1:2000, Sigma). Equal loading was controlled by incubating the membrane with mouse anti-alpha-tubulin antibody (1:10000, Sigma), and goat anti- mouse IgG antibody (1:20000, Pierce).

## Reagents

**Table:** 

**Strains**	**Genotype**	**Available from**
N2		CGC
MQ125	*clk-2 ( qm37 ) *	CGC
SP506	*clk-2 ( mn159 ) *	CGC
MQ742	*clk-2 (qv36* C772Y-A828T)	Hekimi lab
MQ743	*clk-2 (qv37* C772Y-A828T)	Hekimi lab
MQ744	*clk-2 (qv38* C772Y-A828T)	Hekimi lab
MQ745	*clk-2 (qv39* C772Y-A828T)	Hekimi lab
MQ746	*clk-2 (qv40* C772Y-A828V)	Hekimi lab
MQ1037	*clk-2 (qv41* C772Y-A828T)	Hekimi lab
MQ1038	*clk-2 (qv42* C772Y-S859N)	Hekimi lab
MQ1039	*clk-2 (qv43* C772Y-A828T)	Hekimi lab
